# Lactate attenuates PANoptosis and enhances ZBP-1 lactylation in macrophages in acute lung injury

**DOI:** 10.3389/fimmu.2025.1648303

**Published:** 2025-12-17

**Authors:** Dan Wang, Yi Gong, Yehao Chen, Zhefan Wang, Jinxiang Yin, Zhengrui Wang, Xiao Yang, Yizhou Zhang, Jie Huang, Zhengyu Jiang

**Affiliations:** 1 Department of Gynecology and Obstetrics, Changzheng Hospital, Naval Medical University, Shanghai, China; 2 Department of Respiratory and Critical Care Medicine, Huashan Hospital Affiliated to Fudan University, Shanghai, China; 3 Department of Anesthesiology, Naval Medical Center, Naval Medical University, Shanghai, China; 4 Yangpu Senior High School, Shanghai, China; 5 Faculty of Anesthesiology, Changhai Hospital, Naval Medical University, Shanghai, China

**Keywords:** lactate, acute lung injury, ZBP1, PANoptosis, lactylation

## Abstract

**Introduction:**

Acute lung injury (ALI), characterized by excessive inflammation and cell death, is closely associated with ZBP1-mediated PANoptosis, a programmed cell death mechanism. The intrinsic regulatory mechanism of PANoptosis remains poorly investigated.

**Methods:**

In this study, we investigated the protective effects of lactate on lipopolysaccharide (LPS)-induced ALI and its potential regulatory role in ZBP1-mediated PANoptosis.

**Results:**

Our results showed that lactate effectively reduced inflammatory cytokine (TNF-α, IL-6, and IL-1β) and CD11b^+^Gr1^+^ monocyte infiltration in bronchoalveolar lavage fluid and serum. It also alleviated lung pathology and suppressed PANoptosis markers (cleaved caspase-1, GSDMD-p20, phospho-MLKL, and cleaved caspase-3/8). Mechanistically, lactate decreased ZBP1 expression and its interaction with RIPK1 and TRIF while enhancing ZBP1 lactylation. Notably, the ZBP1 agonist CBL0137 reversed the protective effects of lactate. *In vitro* experiments demonstrated that lactate inhibited LPS + 5z7-induced PANoptosis in macrophages by reducing the ZBP1-RIPK1-TRIF interaction and promoting ZBP1 lactylation, which was dependent on MCT1. Thus, our study demonstrated that lactate attenuates ALI by suppressing ZBP1-mediated PANoptosis, possibly through Mct1-related lactylation.

**Conclusion:**

The present study revealed the regulatory role of lactate in inflammation and cell death in ALI and suggested a lactylation-associated therapeutic strategy for ALI.

## Introduction

Acute lung injury (ALI) and acute respiratory distress syndrome (ARDS) are characterized by the abrupt onset and persistent manifestation of acute inflammatory responses (1–4). Pathogen-associated molecular patterns (PAMPs), exemplified by lipopolysaccharides, potently stimulate innate immune cells to release a surge of inflammatory cytokines, serving as the primary instigator of ALI initiation and maintaining a continuous inductive influence throughout its pathophysiological progression (2). This inflammatory cascade not only exacerbates localized pulmonary inflammation but also precipitates a spectrum of pathological alterations, including inflammatory cell infiltration, tissue edema, and cell death (1). Concomitant with this inflammatory response is a metabolic adaptation that facilitates respiratory bursts and augmented energy metabolism, ultimately culminating in substantial lactate production via the Warburg effect (3–5).

Recent advances in ALI pathogenesis research have revealed the critical role of PANoptosis, a programmed cell death mechanism that participates in the progression of lung injury (6–8). This process encompasses various modes of cell death, including necroptosis, pyroptosis, and apoptosis (9). A key regulator of PANoptosis is Z-DNA binding protein 1 (ZBP1), which plays a pivotal role in initiating downstream cell death signaling pathways (10, 11). ZBP1 interacts with RIPK1 through its RHIM domain to facilitate the assembly of the PANoptosis complex, thereby triggering the activation of RIPK3/MLKL-mediated necroptosis, caspase-1/GSDMD-mediated pyroptosis, and caspase-8-mediated apoptosis (11, 12). The appropriate activation of ZBP1 and subsequent cell death are crucial for eliminating pathogens and damaged cells. Conversely, excessive activation of ZBP1 leads to pronounced cellular damage, membrane rupture, and the release of danger-associated molecular patterns (DAMPs), further exacerbating inflammation (13). Consequently, intricate regulatory mechanisms have evolved to finely tune ZBP1 activation, particularly through direct posttranslational modifications.

Recent research has underscored the multifaceted role of lactate, highlighting its role in regulating inflammation, immune cell differentiation, and immunosurveillance via a process known as lactylation, an epigenetic modification that covalently influences its activation, degradation or molecular interaction (3). Lactylation occurs in both histone and nonhistone proteins (14, 15). Histone lactylation of H3 or H4 regulates mRNA transcription, whereas nonhistone lactylation affects metabolism, signal transduction, protein activation, degradation and interactions (8, 14–17). However, whether lactate exerts regulatory effects on ZBP1-mediated PANoptosis and possibly influences the lactylation of ZBP1 remain unknown.

The present study focused on elucidating the effects of intratracheal lactate administration and exploring its potential regulatory role in lipopolysaccharide (LPS)-induced ALI. Our observations revealed notable protective effects of lactate against ALI and its suppression of PANoptosis in lung tissues, which could be abrogated by an agonist of ZBP1. Our data indicated that lactate specifically influences ZBP1 activation in macrophages, enhancing the lactylation of ZBP1 and decreasing its interaction with RIPK1 and Cas8, which may result in the suppression of ZBP1-mediated PANoptosis. Overall, this study identifies a novel protective role for lactate in ALI and elucidates the intrinsic regulatory mechanism of lactate through PANoptosis and lactylation, shedding new light on potential therapeutic interventions.

## Materials and methods

### Animal model

Eight- to ten-week-old male C57BL/6J mice were purchased from Modelorg Inc. (Shanghai, China) and housed in controlled environments maintained at a temperature range of 18–22°C, with a relative humidity of 50–60%, and a 12-hour light–dark cycle. The mice had unrestricted access to standard food and water. Ethical approval for the animal experiments was granted by the Ethical Committee of Changhai Hospital in accordance with the Experimental Animal Regulations of Naval Medical University. To induce acute lung injury (ALI), the mice were initially anesthetized via a mixture of 1.5–2% sevoflurane and oxygen administered via an anesthetic evaporator (Drager, Germany). Subsequently, lipopolysaccharide (LPS) derived from *Escherichia coli* O111:B4 (Sigma, China), at a dose of 10 mg/kg and diluted in 50 µl of PBS, was administered through tracheal intubation. Concomitantly, lactate (Sigma, China) was co-administered intratracheally with LPS at a final concentration of 10 mM, which was diluted in the same 50 µl PBS solution. For the negative control group, an equal volume of sterile PBS without LPS or lactate was administered via the same intratracheal route. Six hours after the injection, blood and lung tissue samples were collected for subsequent analysis. During the 6-hour observation period following the single-dose intratracheal administration, no mortality was recorded in any experimental group (control, LPS, and LPS+lactate groups). All mice maintained stable vital signs without overt signs of respiratory distress, lethargy, or mortality throughout the experimental period.

### Murine macrophage preparation and stimulation

For the preparation of bone marrow-derived macrophages (BMDMs), bone marrow was collected from the femoral tissues of C57BL6J mice flushed with 3 ml of normal saline (NS). After red-blood-cell lysis, the bone marrow cells were resuspended at a concentration of 2–4×10^6^ cells/ml in Dulbecco’s modified Eagle’s medium (DMEM) supplemented with 10% FBS and 30 ng/ml GM-CSF. The culture medium was changed every two days. After 5–6 days of culture, the cells were used for further experiments. Biological replicates were using BMDMS isolated from three independent mice, cultured, treated, and detected separately. To stimulate PANoptosis of macrophages, LPS (10ng/ml) (*E. coli* O111:B4, Sigma, China) and 5Z-7-Oxozeaenol (5z7, 125nM) (12) were simultaneously added, and two hours after the administration, cells were subjected to analysis.

### Cytokine measurement

Cytokines in the serum or supernatant were quantified via enzyme-linked immunosorbent assay (ELISA) kits from R&D Systems (USA) in accordance with the manufacturer’s instructions.

### Lactate dehydrogenase release assay

To assess cell death, the release of lactate dehydrogenase (LDH) into the culture supernatant of macrophages was measured using a commercial LDH Cytotoxicity Assay Kit (e.g., Beyotime Biotechnology, China) following the manufacturer’s instructions. Briefly, after indicated treatment of cells, 50 μL of cell culture supernatant was collected and mixed with an equal volume of LDH reaction solution in a 96-well plate. The mixture was incubated at 37°C in the dark for 30 minutes, and the absorbance was measured at 490 nm using a microplate reader (Bio-Rad, USA). The percentage of LDH release (an indicator of cell death) was calculated as follows: (Experimental absorbance - Blank absorbance)/(Maximum LDH release absorbance - Blank absorbance) × 100%. Maximum LDH release was achieved by lysing control cells with the lysis buffer provided in the kit. All experiments were performed in replicates.

### Immunoblotting and immunoprecipitation

The cells were lysed via radioimmunoprecipitation assay (RIPA) buffer (Beyotime, China), and protein concentrations were determined via a BCA assay (Thermo, China). The total protein was subjected to SDS–PAGE and transferred onto polyvinylidene fluoride (PVDF) membranes (Merck, Germany), followed by blocking with 5% nonfat dry milk in phosphate-buffered saline with Tween (PBST) at pH 7.5. The membranes were subsequently immunoblotted with primary antibodies for 4 hours and then incubated with horseradish peroxidase (HRP)-conjugated secondary antibodies (Cell Signaling Technology, USA). The antibodies and reagents utilized in this study are listed in **Supplementary Table 1**. The protein bands were detected via an enhanced chemiluminescence kit (Pierce, USA) and visualized via a ChemiDoc XRS+ system (Bio-Rad, China).

For the immunoprecipitation assay, the cells were lysed in buffer consisting of 20 mM PIPES (pH 6.8), 1% Triton X-100, 150 mM NaCl, 150 mM sucrose, 0.2% sodium deoxycholate, 500 μM EDTA, and protease inhibitors on ice for 5 minutes. The samples were then centrifuged, and the supernatants were diluted to a concentration of 2 μg/mL in a dilution buffer comprising 20 mM PIPES (pH 6.8), 1% Triton X-100, 150 mM NaCl, 150 mM sucrose, 2.5 mM MgCl2, and 2.5 mM MnCl2. Primary antibody-conjugated protein A beads (Sigma, USA) were incubated at 4°C for two hours and washed with dilution buffer. Immunoblot analysis was conducted via the aforementioned methods.

### siRNA knockdown

siRNA-mediated knockdown was performed in PMs or BMDMs. The cells were cultivated in half of the culture volume of FBS-free medium and transfected with 3 ng/ml siRNA or control vector using INTERFEREin (Invitrogen, USA) for 6 hours. Thereafter, the remaining half of the culture medium was added, and the cells were further cultivated for 48 hours. Following a 48-hour interference period, the cells were subjected to subsequent experimental procedures.

### Flow cytometry

The cells were stained with primary antibodies at room temperature for 30 minutes, washed three times, and then incubated with the indicated antibodies at 4°C for 15 minutes. The cells were washed, resuspended in washing buffer, and analyzed. The fluorescence data for 10^^5^ events from each sample were acquired on a FACS LSR II (BD Bioscience, USA) and analyzed via FlowJo software (Tomy Digital Biology Co., Ltd., Japan).

### Statistical analysis

The obtained results were subjected to statistical analysis via Student’s t test or analysis of variance (one-way or two-way ANOVA) with appropriate multiple comparisons, as applicable, via Prism software (version 10.0; GraphPad Software, Inc.). A p value of less than 0.05 was considered statistically significant.

## Results

### Lactate attenuates inflammation and pathology in the lungs in LPS-induced ALI

**Figure 1:**
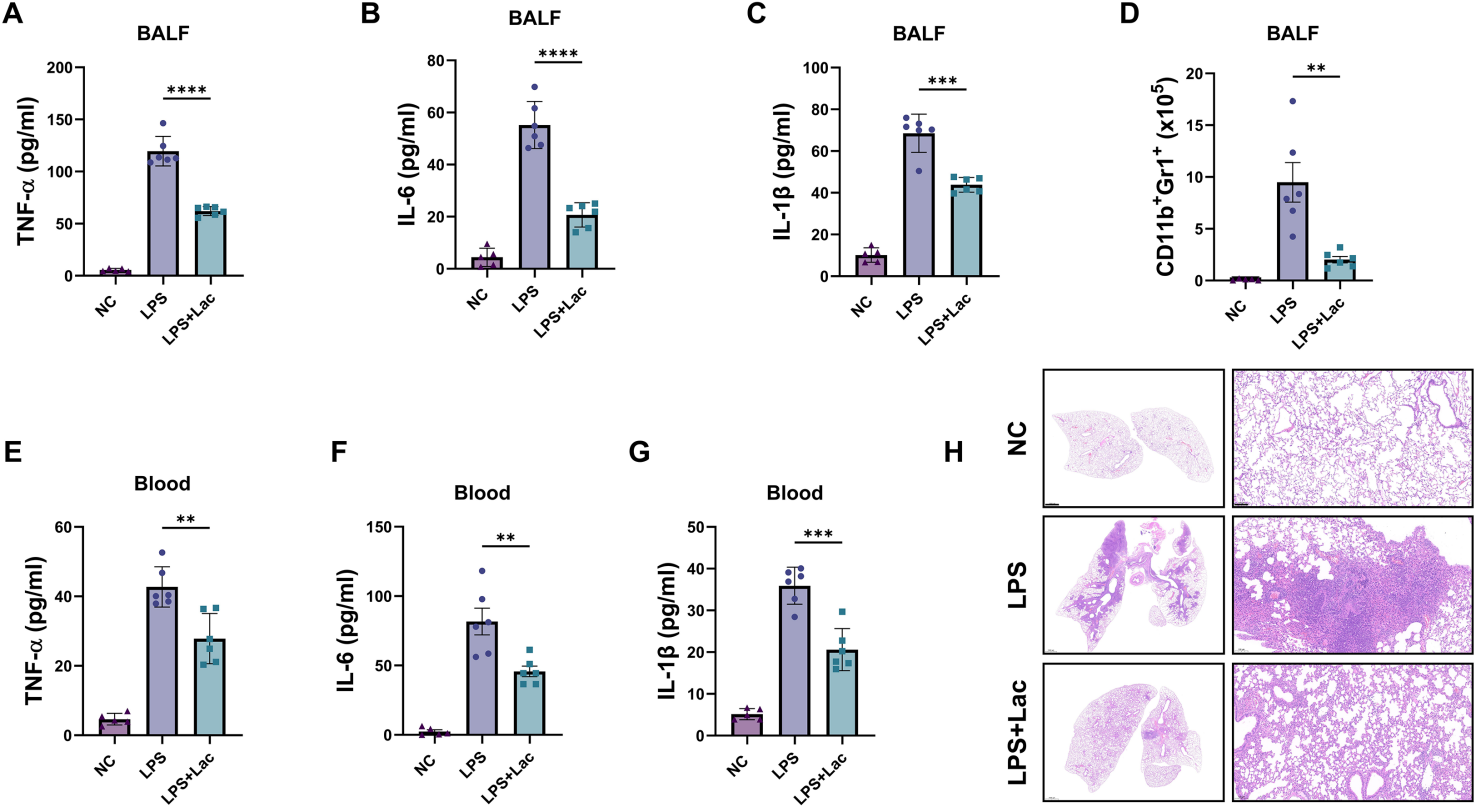
Lactate attenuates inflammation and pathology in the lungs in LPS-induced ALI. **(A–C)** Concentrations of TNF-α **(A)**, IL-6 **(B)**, and IL-1β **(C)** in bronchoalveolar lavage fluid (BALF) measured via ELISA. The data are presented as the means ± SDs (n=6). **(D)** Flow cytometric analysis of CD11b^+^Gr-1^+^ cells in the BALF. The data are presented as the means ± SDs (n=6). **(E–G)** Concentrations of TNF-α **(E)**, IL-6 **(F)**, and IL-1β **(G)** in the blood were measured via ELISA. The data are presented as the means ± SDs (n=6). **(H)** Representative histopathological sections of lung tissue stained with hematoxylin and eosin (H&E). Scale bars, 1mm (left) and 100 μm (right). All the data were analyzed via one-way ANOVA followed by Tukey’s multiple comparison test. **P < 0.01, ***P < 0.001, ****P < 0.0001 *vs*. the NC group; n=biological replicates.

To evaluate the effects of lactate in LPS-induced acute lung injury, we injected lactate through tracheal intubation together with LPS. Six hours after the injection, we analyzed inflammatory infiltration and cytokine levels in the BALF, serum cytokine levels and pathological changes in the lung tissues. We found that lactate attenuated the infiltration of nearly half of the inflammatory cytokines, including TNF-alpha, IL-6 and IL-1beta (**Figures 1A–C**), and decreased the infiltration of CD11b^+^Gr1^+^ monocytes (**Figure 1D**) in the BALF. Similarly, the serum levels of these cytokines were also decreased in the lactate-treated mice (**Figures 1E–G**). Pathology through HE staining of lung tissues revealed significant alleviation of inflammation in lung tissues and that LPS induced inflammatory infiltration and lung consolidation centered on the bronchus, whereas lactate treatment significantly reduced this pathology (**Figure 1H**). Together, these results indicate that intratracheal lactate attenuates lung inflammation in LPS-induced ALI.

### Lactate alleviates inflammatory signaling and cell death in lung tissues in LPS-induced ALI

**Figure 2:**
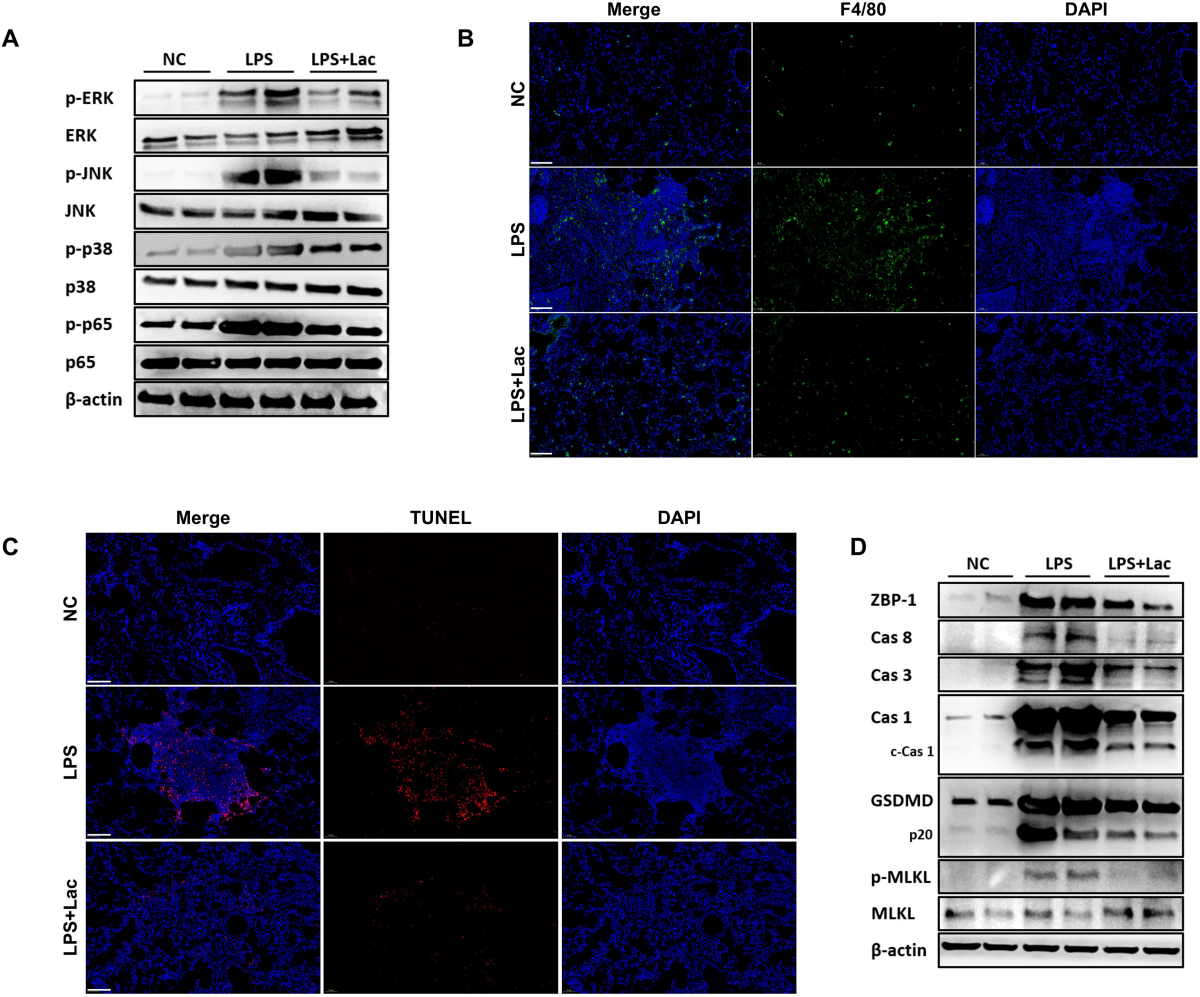
Lactate alleviates inflammatory signaling and cell death in lung tissues in LPS-induced ALI. **(A)** Western blot analysis of the protein levels of p-ERK, ERK, p-JNK, JNK, p-p38, p38, p-p65, and p65 in the lung tissues of the NC (negative control), intratracheally administered LPS, or LPS+Lactate (LPS+Lac) groups; β-actin was used as an internal control. **(B)** Representative immunofluorescence images showing F4/80 (green) and DAPI (blue) staining in the lung tissues of the indicated groups. The merged images are shown on the left. Scale bars, 100 μm; **(C)** TUNEL assay (red) and DAPI (blue) staining were used to detect cell death in lung tissues. Merged images are shown on the left. Scale bars, 100 μm; **(D)** Western blot analysis of the protein levels of ZBP-1, Cas 8, Cas 3, Cas 1, c-Cas 1, GSDMD, p20, p-MLKL, and MLKL in lung tissues. β-actin was used as an internal control.

The vicious cycle of inflammatory infiltration-mediated cellular injury and subsequent tissue damage plays a critical role in exacerbating lung injury. Our experiments revealed that LPS challenge markedly activated key inflammatory signaling pathways in lung tissues, as evidenced by increased phosphorylation levels of p65, p38, JNK, and ERK (p < 0.05). Notably, lactate treatment effectively suppressed these LPS-induced phosphorylation events (**Figure 2A**). Immunohistochemical analysis further confirmed the anti-inflammatory properties of lactate, which significantly reduced macrophage infiltration (F4/80+ cells) in LPS-challenged lungs following lactate administration (**Figure 2B**). Given the intricate relationship between inflammation and programmed cell death, we first adopted TUNEL analysis to determine the degree of cell death in lung tissues. LPS administration increased the degree of death of lung tissues, whereas lactate treatment reduced the degree of injury (**Figure 2C**). These results indicated that lactate alleviated inflammatory infiltration and cell death in lung tissues in LPS-induced ALI.

Recent studies have shown that programmed cell death, characterized by apoptosis, necroptosis and pyroptosis, plays a pivotal role in the progression of tissue damage in ALI and can be regulated by the upstream molecule ZBP1 (6, 18). We then examined apoptosis (mediated by Caspase-3/8), necroptosis (marked by phosphor-MLKL), and pyroptosis (characterized by Caspase-1 activation and GSDMD cleavage). We demonstrated that lactate treatment broadly inhibited the expression of cell death markers, significantly reducing the levels of GSDMD-p20 (pyroptosis), phospho-MLKL (necroptosis), and Caspase-3 (apoptosis) (**Figure 2D**). Moreover, lactate suppressed upstream regulators, including Caspase-8 and cleaved Caspase-1 (c-Cas-1), which control MLKL activation and GSDMD processing, respectively.

Collectively, our data demonstrate that lactate exerts dual protective effects on LPS-induced acute lung injury by simultaneously mitigating inflammatory signaling activation (through the p65/p38/JNK/ERK pathways) and interrupting the PANoptosis cascade, possibly via ZBP1-dependent mechanisms.

### Lactate attenuates LPS-induced ALI through ZBP1-mediated PANoptosis

**Figure 3:**
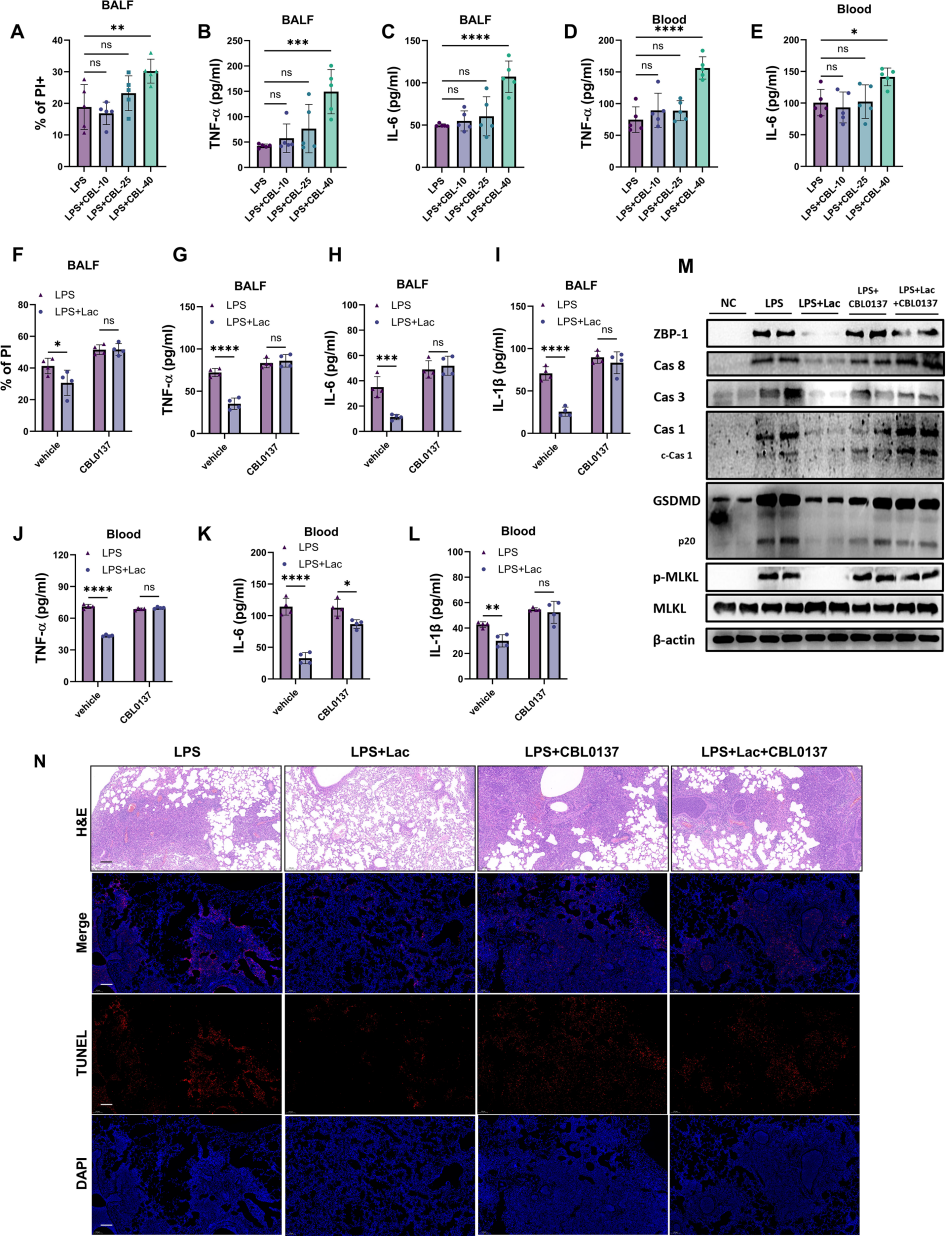
Lactate attenuates LPS-induced ALI through ZBP1-mediated PANoptosis.**(A)** Flow cytometric analysis of PI^+^ cells in bronchoalveolar lavage fluid (BALF). The data are presented as the means ± SDs (n=5). **(B–E)** Concentrations of TNF-α and IL-6 in the BALF **(B, C)** and blood **(D, E)** were measured via ELISA. The data are presented as the means ± SDs (n=6). **(F–I)** PI^+^ cells and concentrations of TNF-α, IL-6 and IL-1β in BALF of mice pre-treated with CBL0137 (40mg/kg) for 1h and then stimulated with intratracheal LPS. The data are presented as the means ± SDs (n=4). **(J–L)** Concentrations of TNF-α, IL-6 and IL-1β in blood of mice pre-treated with CBL0137 (40mg/kg) for 1h and then stimulated with intratracheal LPS. The data are presented as the means ± SDs (n=4). **(M)** Western blot analysis of ZBP-1, Cas 8, Cas 3, Cas 1, c-Cas 1, GSDMD, p20, p-MLKL, and MLKL protein levels in lung tissues of mice pre-treated with CBL0137 (40mg/kg) for 1h and then stimulated with intratracheal LPS. β-actin was used as an internal control. **(N)** Representative histopathological sections of lung tissue stained with hematoxylin and eosin (H&E). Scale bars, 100 μm. Representative immunofluorescence images showing TUNEL (red) and DAPI (blue) staining of lung tissue. Scale bars, 50 μm. All the data were analyzed via one-way ANOVA followed by Tukey’s multiple comparison test. *P < 0.05, **P < 0.01, ***P < 0.001, ****P < 0.0001 *vs*. the vehicle group; ns, not significant; n=biological replicates.

**Figure 4:**
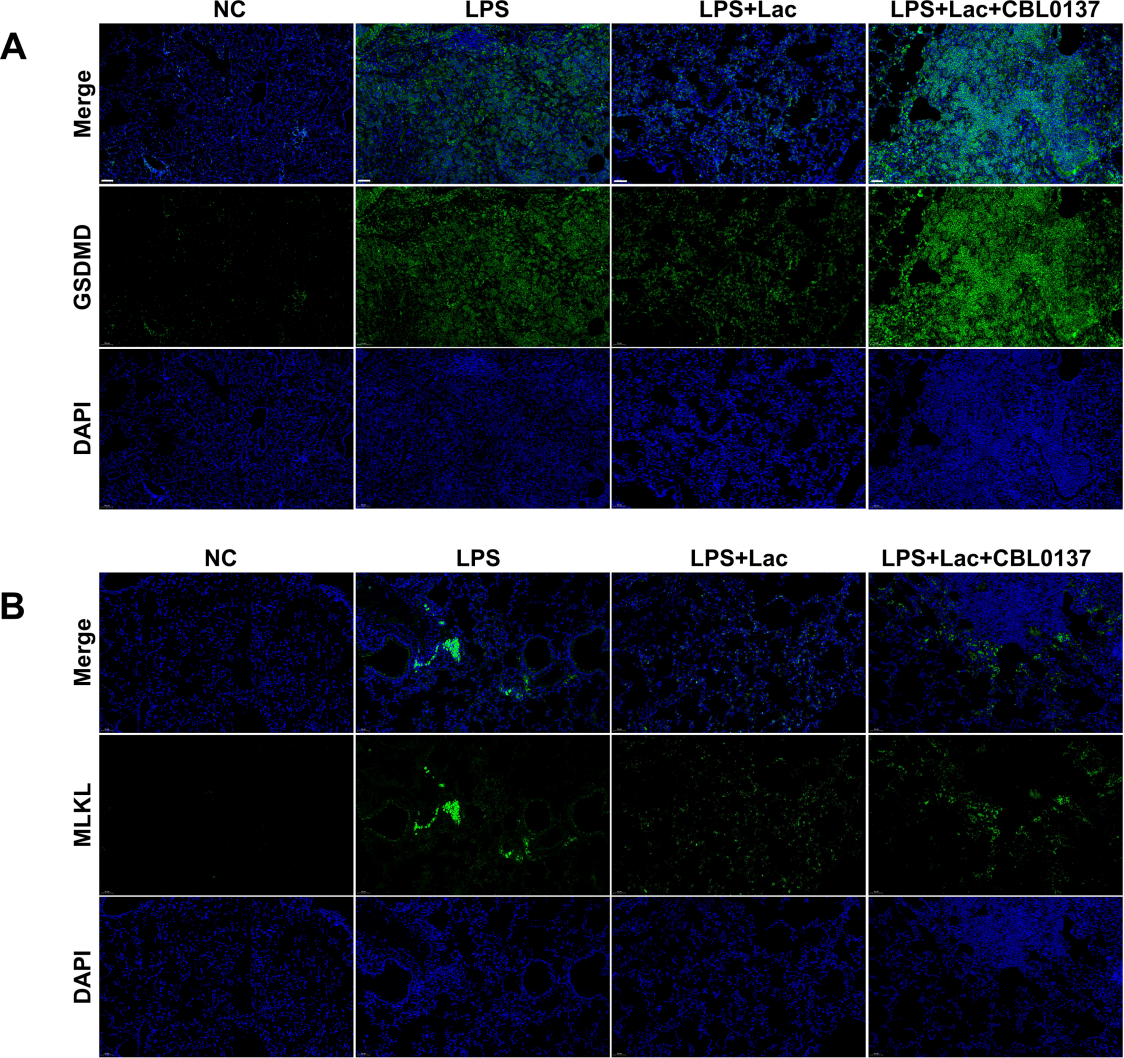
Lactate attenuates GSDMD and MLKL activation in lung tissues in LPS-induced ALI. **(A)** Representative immunofluorescence images showing GSDMD (green) and DAPI (blue) staining in lung tissue. The merged images are shown at the top. Scale bars, 100 μm. **(B)** Representative immunofluorescence images showing MLKL (green) and DAPI (blue) staining in lung tissue. The merged images are shown at the top. Scale bars, 100 μm. NC, negative control, cells without any treatment or stimulation; LPS, lipopolysaccharide; Lac, lactate; CBL0137: ZBP1 agonist.

The above results indicated that lactate suppressed ZBP1 expression and the downstream activation of Caspase-8 and Caspase-1 in the lung tissues of patients with LPS-induced ALI (**Figure 2D**). These results suggest that lactate may suppress PANoptosis, including pyroptosis, apoptosis and necroptosis, through the inhibition of ZBP1. To investigate the essential role of ZBP1 in lactate treatment, First, we examined the *in vivo* concentration of CBL capable of activating ZBP1. Our findings indicated that at a dose of 40 mg/kg, CBL exacerbated lung injury, elevated levels of inflammatory cytokines, and induced cell death in bronchoalveolar lavage fluid (BALF) (**Figures 3A–E**), suggesting that this concentration is sufficient to trigger ZBP1-mediated PANoptosis. We then applied this dosage to assess the role of ZBP1 in the effect of lactate. The results showed that CBL0137 abrogated the protective effects of lactate. TNF-alpha, IL-6, and IL-1beta levels and cell death in the BALF did not differ between the LPS and LPS+lacate groups in the presence of CBL0137 (**Figures 3F–I**). Moreover, the lactate-mediated attenuation of inflammatory cytokines in the serum was also abrogated by CBL0137 in LPS-induced ALI (**Figures 3J–L**). Western blots of lung tissues also revealed that CBL0137 abrogated the lactate-mediated downregulation of ZBP1, as well as caspase 3/8, and the activation of Caspase 1, GSDMD and phospho-MLKL (**Figure 3M**), indicating that lactate alleviated PANoptosis through the suppression of ZBP1. Pathological analysis and TUNEL immunofluorescence also revealed that CBL0137 treatment reversed the protective effects of lactate (**Figure 3N**). We also analyzed the immunohistochemistry of GSDMD and MLKL *in vivo*. Lactate administration significantly decreased the activation of GSDMD and MLKL in lung tissues, whereas CBL0137 reversed the downregulation of these two molecules by lactate (**Figures 4A, B**). These results further support the essential role of ZBP-1-mediated PANoptosis in the protective effects of lactate in ALI. These results suggest that lactate attenuates PANoptosis in LPS-induced ALI through the suppression of ZBP1.

### Lactate suppressed ZBP1-mediated PANoptosis and promoted ZBP1 lactylation in macrophages

The broad inhibition of PANoptosis by lactate prompted us to investigate the effects of lactate on ZBP1, a coordinating regulator of these three types of programmed cell death, which is termed PANoptosis. The above results indicated that lactate significantly attenuated ZBP1 expression in lung tissues (**Figure 2D**) and that increasing ZBP1 reversed the lactate-mediated attenuation of PANoptosis (**Figure 3**). Inflammation of lung tissues in ALI is initiated by the activation of innate immune cells and subsequent inflammatory cytokine production (19). Recent studies have shown that PANoptosis in macrophages plays a crucial role in exacerbating inflammation in multiple organ injury and that macrophage activation and cell death participate in the pathophysiology of ALI (6, 9, 20, 21). Therefore, we investigated the effects of lactate on PANoptosis in macrophages.

**Figure 5:**
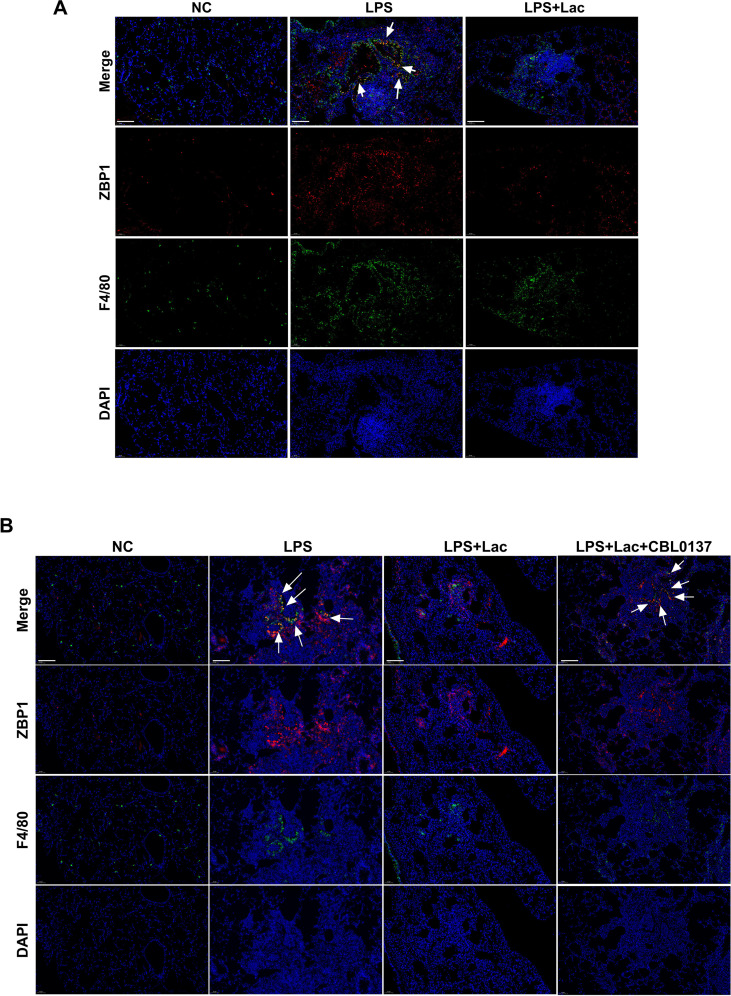
Lactate suppresses ZBP1 activation in macrophages in lung tissues in LPS-induced ALI. **(A)** Representative immunofluorescence images showing ZBP1 (red), F4/80 (green), and DAPI (blue) staining in lung tissue. The merged images are shown at the top. The arrows indicate colocalization in the ZBP1^+^F480^+^ region. Scale bars, 100 μm. **(B)** Representative immunofluorescence images showing ZBP1 (red), F4/80 (green), and DAPI (blue) staining in lung tissue. The merged images are shown at the top. The arrows indicate colocalization in the ZBP1^+^F480^+^ region. Scale bars, 100 μm. NC, negative control, cells without any treatment or stimulation; LPS, lipopolysaccharide; Lac, lactate (10mM); CBL0137: ZBP1 agonist. n=biological replicates.

Indeed, immunofluorescence analysis provided spatial details of this phenomenon, demonstrating that the LPS-induced colocalization of the macrophage marker F4/80 with ZBP1 was dramatically reduced by lactate treatment (**Figure 5A**, white arrows). CBL0137 activated ZBP1 in macrophages (**Figure 5B**, white arrows). These findings suggest that lactate specifically modulates ZBP1 expression in infiltrating macrophages.

**Figure 6:**
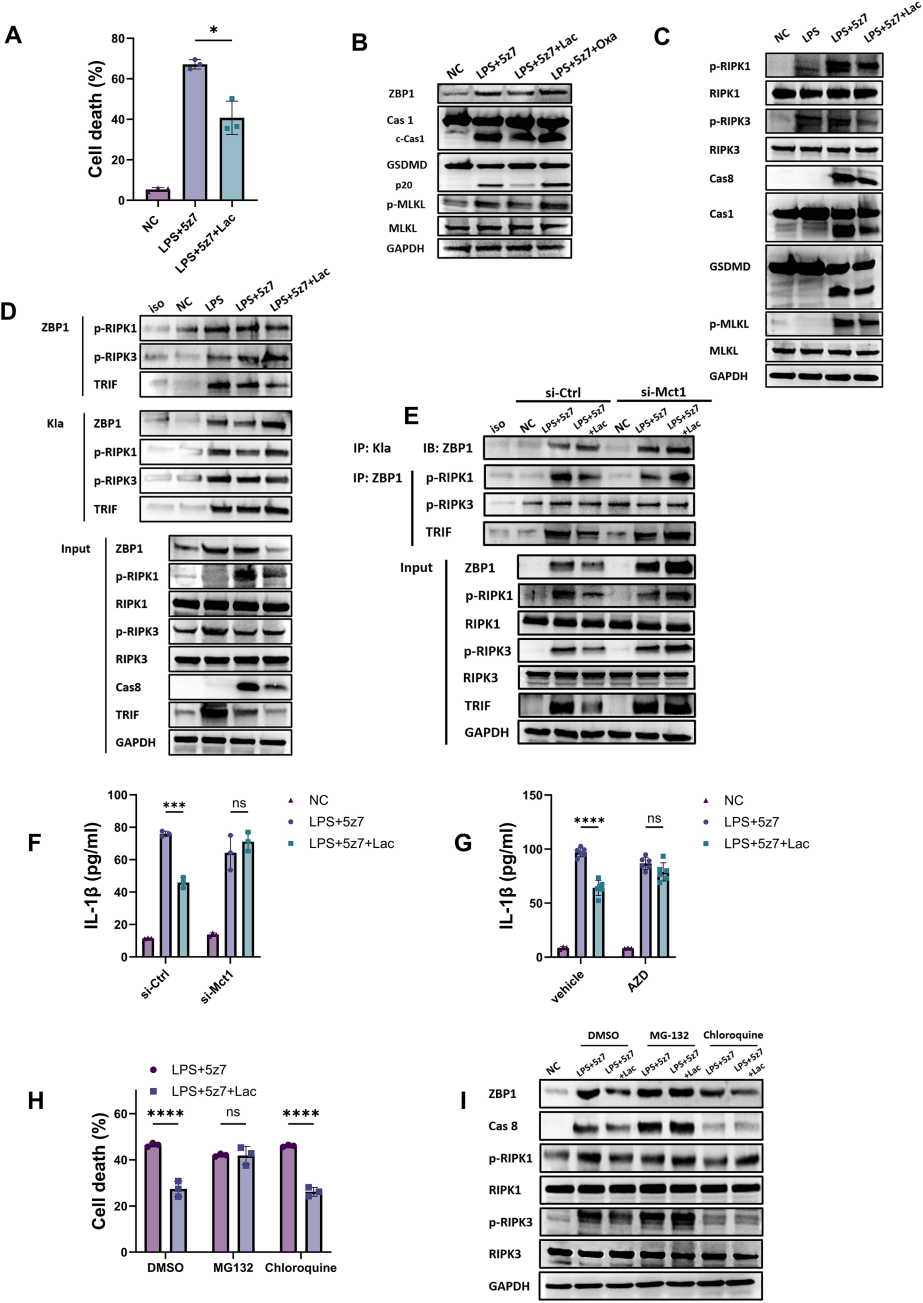
Lactate suppressed ZBP1-mediated PANoptosis and promoted ZBP1 lactylation in macrophages. **(A)** Cell death analyzed by PI^+^ of flow cytometry of BMDMs (n=3). **(B)** Western blot analysis of ZBP1, Cas 1, c-Cas 1, GSDMD, p20, p-MLKL, and MLKL protein levels in macrophages treated with NC, LPS + 5z7, LPS + 5z7+Lac, or LPS + 5z7+Oxa. GAPDH was used as an internal control. **(C)** Western blot analysis of p-RIPK1, RIPK1, p-RIPK3, RIPK3, Cas8, Cas1 and GSDMD protein levels in macrophages treated with NC, LPS + 5z7, or LPS + 5z7+Lac. GAPDH was used as an internal control. **(D)** Coimmunoprecipitation assay of ZBP1 and lactylation protein (Klac) and immunoblotting with p-RIPK3, p-RIPK1, ZBP 1 and TRIF antibodies in macrophages treated with NC, LPS, LPS + 5z7, or LPS + 5z7+Lac. **(E)** Coimmunoprecipitation assay of ZBP1 and Klac and immunoblotting with p-RIPK3, p-RIPK1, ZBP 1 and TRIF antibodies in macrophages treated with si-Ctrl or si-Mct1 for 48 hours, followed by LPS + 5z7+Lac treatment. **(F)** Concentrations of IL-1β in the supernatants of macrophages treated with si-Ctrl or si-Mct1 for 48 hours, followed by LPS + 5z7+Lac treatment. The data are presented as the means ± SDs (n=3). **(G)** Concentrations of IL-1β in the supernatants of macrophages treated with vehicle or AZD. The data are presented as the means ± SDs (n=3). **(H, I)** Cell death analysis by LDH release measurement **(H)** and western blot **(I)** of macrophage pretreated with MG-132 or chloroquine for 30min and then stimulated with LPS + 5z7 or LPS + 5z7+Lac (n=3). NC: negative control, cells without any treatment or stimulation; *P < 0.05, ***P < 0.001, ****P < 0.0001 *vs*. the NC group; ns, not significant. n=biological replicates.

We next asked how lactate regulates ZBP1 in macrophages. We adopted a well-studied model of PANoptosis in macrophages through the coadministration of LPS and 5Z-7-oxozeaenol (5z7), a selective TAK1 inhibitor (12, 22). *In vitro* analysis demonstrated that LPS + 5z7 treatment induced significant cell death with the activation of ZBP1 and downstream Cas1, GSDMD and MLKL. However, lactate administration suppressed the LPS + 5z7-induced cell death and the activation of ZBP1 and subsequent pyroptosis and necroptosis, which were marked by cleaved caspase 1 (c-Cas1), GSDMD-p20 and phospho-MLKL (**Figures 6A, B**). In contrast, the administration of oxamate, a selective inhibitor of LDH-A that inhibits intrinsic lactate production (5, 14), increased ZBP1 expression, as did the activation of GSDMD-p20 and phospho-MLKL (**Figure 6B**), suggesting that intrinsic lactate also moderately attenuated ZBP1 activation.

We then asked how lactate influences the activation of ZBP1 and subsequent PANoptosis. Research has revealed that ZBP1 can interact with RIPK1 and TRIF through their RHIM domain and initiate the formation of the TRIFosome complex, which eventually activates Caspase1-dependent pyroptosis, caspase8-dependent apoptosis and RIPK3/MLKL-mediated necroptosis (12, 23, 24). We found that lactate administration suppressed the activation of RIPK1 and RIPK3 (p-RIPK1 and p-RIPK3), as well as Cas8 and TRIF, both of which participate in PANoptosome formation (**Figure 6C**). These data suggest that lactate may suppress the formation of PANoptosomes, which is dependent on the ZBP1-RIPK1 interaction. Furthermore, immunoprecipitation analysis revealed that lactate administration suppressed the interaction of ZBP1 with p-RIPK1 and TRIF (**Figure 6D**). We also observed an enhanced interaction of p-RIPK3 with ZBP1 (**Figure 6D**), which might be a compensatory mechanism of RIPK1 and RIPK3, as both molecules contain the RHIM domain. These results indicated that lactate administration suppressed the interaction between ZBP1 and RIPK1.

To investigate how lactate suppresses the ZBP1-RIPK1 interaction, we analyzed the possible role of lactylation, which has been reported to influence the molecular interaction (25, 26). Interestingly, only ZBP1 and RIPK1 presented increased lactylation, whereas TRIF and RIPK3 were unaltered or decreased (**Figure 6D**). These results suggested that lactate administration enhanced the lactylation of ZBP1 and RIPK1, possibly influencing the ZBP1-RIPK1 interaction. To investigate the role of lactylation in ZBP1 function, we knocked down Mct1 (3, 27, 28), a previously reported membrane channel that crucially mediates lactylation. We found that Mct1 knockdown abolished the increase in ZBP1 lactylation (**Figure 6E**). Moreover, the decreased interaction between ZBP1 and RIPK1 mediated by lactate administration was also abrogated by Mct1 knockdown, suggesting that Mct1-mediated lactylation is crucial for the lactate-induced suppression of ZBP1-PANoptosis. We further demonstrated that the production of IL-1beta, a feature of LPS + 5z7-induced PANoptosis (12), was suppressed by lactate, whereas siRNA knockdown or pharmaceutical inhibition of Mct1 abrogated the lactate-induced suppression of IL-1beta (**Figures 6F, G**). Thus, these results suggested that lactate-induced PANoptosis was dependent on Mct1, possibly through Mct1-mediated lactylation.

We then investigated how lactate promoted the degradation of ZBP1. Previous study indicated proteins lactylation targeted to proteasomal degradation (29). Therefore, we applied the proteasome inhibitor, MG-132, to analyze the effects of lactate. We found MG-132, but not chloroquine, the autophagy inhibitor that also participates in protein degradation, abrogated the effects of lactate in reducing cell death as well as the downregulation of ZBP1 and PANoptosome (**Figures 6H, I**), suggesting lactate promote ZBP1 proteasomal degradation through lactylation.

Thus, our data indicated that lactate administration enhanced the lactylation of ZBP1 and RIPK1 and decreased the interaction between RIPK1 and ZBP1. We also showed that Mct1 was crucial for lactate-mediated suppression of ZBP1-PANoptosis.

## Discussion

In the present study, we demonstrated that lactate protected against LPS-induced acute lung injury through attenuating ZBP1-mediated PANoptosis. We found that lactate downregulated the expression of markers of pyroptosis, necroptosis and apoptosis induced by ZBP1 activation, which could be abrogated by a ZBP1 agonist. Specifically, we observed that lactate inhibited ZBP1 activation in macrophages during ALI and suppressed PANoptosis in macrophages *in vitro*. Lactate administration decreased the interaction of ZBP1 with RIPK1 and caspase 8 and enhanced the lactylation of ZBP1. Knockdown of Mct1, an essential transporter for lactylation, reversed the attenuation of PANoptosis by lactate in macrophages.

ALI/ARDS is associated with uncontrolled inflammation, cell death, immune cell infiltration and cytokine production (1). Among these pathogeneses, increased lactate levels both regionally and in circulation have been observed and reported to have diverse functions, including metabolic reprogramming, inflammatory regulation and epigenetic modification (1–4). In our study, we found that intratracheal lactate administration had protective effects on ALI, attenuated inflammatory cytokine production in both BALF and serum, suppressed inflammatory infiltration and suppressed PANoptosis in lung tissues. The decreased cytokine production and preferential downregulation of ZBP1 in macrophages (**Figure 5**) suggested a crucial role of macrophages in the protective effects of lactate during ALI. Our results showed lactate reduced LPS-induced phosphorylation of NF-κB p65 and MAPKs (ERK, JNK, p38) (**Figure 2A**), which aligns with previous studies reporting lactate’s inhibition of these pathways to alleviate inflammation (30, 31). Though attenuated NF-κB and MAPK signaling provide essential role in the protective effects of lactate, we highlight lactate exerts ALI protection not only via dampening inflammation but also by restraining ZBP1-dependent PANoptosis, offering a more comprehensive protective mechanism. Specifically, we found that lactate decreased ZBP1 activation in macrophages in lung tissues and that ZBP1 agonism via CBL0137 reversed the protective effects of lactate, suggesting that ZBP1 might be a pivotal target of lactate in macrophages. Notably, it is acknowledged that CBL0137 has been reported to interact with the FACT complex (32), which may potentially modulate pro-inflammatory and cell death pathways. The potential off-target effects could also participate in the process of ALI. Therefore, we conducted multiple lines of experiments to support that the abrogation of lactate’s protective effects by CBL0137 is primarily mediated through ZBP1 activation rather than off-target interactions. The data of reduced ZBP1 expression, enhanced ZBP1 lactylation, and disrupted ZBP1-RIPK1/TRIF interaction, as well as the *in vivo* concentration of CBL0137 (40 mg/kg) validated to specifically activate ZBP1-dependent PANoptosis (**Supplementary Figure 1**) without causing non-specific cytotoxicity supported the present conclusion. However, specific knockout models in future study may provide key evidence. Thus, while CBL0137 may have potential off-target interactions, the consistent molecular and phenotypic data strongly indicate that ZBP1 is the primary target mediating lactate’s protective effects against ALI.

These findings highlight the novel role of lactate in the pathophysiology of ALI and suggest the intrinsic interplay of the recruitment of innate immune cells during ALI and the production of lactate in response to pathogens or stimuli. The increased lactate level suppresses PANoptosis in macrophages, preventing excessive cellular injury and the release of DAMPs and thus tuning inflammatory responses. To our knowledge, this is the first study highlighting the crucial role of lactate in regulating ZBP1 and PANoptosis in ALI. However, this phenomenon may be cell-specific or produce different effects due to variations in the local concentration of lactate. Therefore, detailed studies are still needed to address this question.

ZBP1-mediated PANoptosis is an extensive form of cell death, including pyroptosis, apoptosis and necroptosis, which is initiated by ZBP1 activation (10, 11, 20, 33–35). A previous study revealed the essential interaction of ZBP1 and RIPK1 in PANoptosis in *Yersinia pseudotuberculosis* infection and revealed that ZBP1 constitutively binds with RIPK1 to initiate the TRIFosome, a newly identified complex containing ZBP1, TRIF, RIPK1, FADD and caspase 8 that induces PANoptosis in Yersinia infection or LPS+TAK1 inhibition (5z7) treatment (11, 12, 22–24). TAK1 inhibition using 5z7 combined with LPS induces significant cell death that specifically resulted from PANoptosis, while LPS or 5z7 along could not induce cell death *in vitro (*12, 36). Our data is in accordance with this phenomenon (**Supplementary Figure 1D**). However, we found a unique phenomenon in which lactate treatment downregulated the interaction of ZBP1 with RIPK1 and TRIF, resulting in suppressed activation of p-MLKL, caspase-1/GSDMD and caspase-8. Interestingly, the decreased interaction may induce subsequent degradation of molecules, as we noted that lactate also decreased the protein levels of ZBP1, RIPK1 and TRIF. Thus, our study highlighted the essential role of the ZBP1-RIPK1-TRIF interaction in PANoptosis and the unique mechanism by which lactate attenuates the interaction of these three molecules.

By investigating the possible mechanism by which lactate regulates ZBP1 activation, we found that lactate enhances the lactylation of ZBP1. Protein lactylation has recently been identified as a new posttranslational modification that regulates protein interactions, signal transduction and cellular metabolism (14, 17, 26). To our knowledge, this is the first study demonstrating that lactate can enhance the lactylation of ZBP1 and may thus influence its function in macrophages. The increased lactylation of ZBP1 and RIPK1 in our study, as well as the decreased interaction mentioned above, may induce protein degradation, which has been well characterized in previous studies (8, 16, 26). To investigate the essential role of ZBP1 lactylation in the effects of lactate, we used siRNA-mediated knockdown and pharmaceutical inhibition of Mct1, a previously reported channel that is essential for exogenous lactate-mediated lactylation (3, 27, 28, 37). Knockdown or pharmaceutical inhibition both abrogated the attenuation of markers of ZBP1-mediated PANoptosis, suggesting that lactylation is essential for lactate regulation of ZBP1-PANoptosis in macrophages. We further showed the lactylation-induced proteasomal degradation is crucial for lactate-mediated ZBP1 downregulation. Although we did not screen out the lactyl-transferase(s) and/or de-lactylase(s) acting on ZBP1, which require large-scale interactome and enzymatic screens that are beyond the scope of the present study, our present data provides a promising direction for ZBP1 lactylation in inflammatory responses and PANoptosis in macrophages. Research on ZBP1 modifications may provide specific targets for translational applications.

Taken together, the results of our study demonstrated that intratracheal lactate has protective effects on murine ALI, improving pathological alterations and attenuating inflammatory cytokine production and cell death in lung tissues. Lactate ameliorated PANoptosis in lung tissues and suppressed ZBP1 activation in macrophages both *in vitro* and *in vivo*. Our data also suggested that lactate may regulate ZBP1 activation through lactylation and subsequently decrease the interaction with RIPK1 and TRIF. Our study revealed a crucial role of lactate in the regulation of ZBP1-mediated PANoptosis and ZBP1 lactylation in ALI, providing new insights into the function of lactate and translational targets in future applications.

## Data Availability

The raw data supporting the conclusions of this article will be made available by the authors, without undue reservation.

## Supplementary material

Supplementary Figure 1 **(A, B)** TNF-alpha and IL-6 of BMDMs pre-treated with CBL0137 at indicated concentration and then stimulated with LPS + 5z7 for 2h (n=3). **(C)** Cell death analyzed by PI^+^ of flow cytometry of BMDMs pre-treated with CBL0137 at indicated concentration and then stimulated with LPS + 5z7 for 2h. **(D)** Cell death analyzed by LDH and IL-1β in supernatant of BMDMs treated with indicated molecules for 2 hours; NC: negative control, cells without any treatment or stimulation; (n=3). *P < 0.05, **P < 0.01, ***P < 0.001, ****P < 0.0001 *vs*. the NC group; ns, not significant. n=biological replicates.
