# Supplementary material for: Lactate attenuates PANoptosis and enhances ZBP-1 lactylation in macrophages in acute lung injury
Source: Front Immunol. 2025 Dec 17;16:1648303. doi: 10.3389/fimmu.2025.1648303 (PMC12754177; doi:10.3389/fimmu.2025.1648303)
Supplement: Supplementary file 2 [file Table1.docx]

**Supplementary Table 1 Antibodies in the study**

| **Antibodies** | **Source** | **Lot number** |
| --- | --- | --- |
| ZBP1 | Abcam | ab227937 |
| Caspase-1 | CST | #22915 |
| Caspase-3 | CST | #9662 |
| cleaved Caspase-3 (Asp175) | CST | #9661 |
| Caspase-8 | CST | #4790 |
| GSDMD | CST | #96458 |
| RIPK1 | CST | #3049 |
| p-RIPK1 (Ser166) | CST | #31121 |
| RIPK3 | CST | #13526 |
| p-RIPK3 (Ser227) | CST | #93653 |
| MLKL | CST | #37707 |
| p-MLKL (Ser345) | CST | #74637 |
| TRIF | Abcam | ab183794 |
| p-ERK1/2 (Thr202/Tyr204) | CST | #4370 |
| ERK1/2 | CST | #4695 |
| p-JNK (Thr183/Tyr185) | CST | #4668 |
| JNK | CST | #9252 |
| p-p38 (Thr180/Tyr182) | CST | #4511 |
| p38 | CST | #9212 |
| p-NF-κB p65 (Ser536) | CST | #3033 |
| NF-κB p65 | CST | #8242 |
| β-actin | CST | #3700 |
| GAPDH | CST | #2118 |
